# Use of three-dimensional computed tomography overlay for real-time cryoballoon ablation in atrial fibrillation reduces radiation dose and contrast dye

**DOI:** 10.1007/s12471-017-0962-7

**Published:** 2017-02-15

**Authors:** B. Oude Velthuis, M. Molenaar, H. G. Reinhart Dorman, J. Y. Stevenhagen, M. F. Scholten, J. van der Palen, J. M. van Opstal

**Affiliations:** 1 Thorax Centre Twente, Medisch Spectrum Twente, Enschede, The Netherlands; 2 Department of Cardiology, Bravis Ziekenhuis, Roosendaal, The Netherlands; 3 Department of Research methodology, Methods and Data Analysis, University of Twente, Enschede, The Netherlands; 4 Medical School Twente, Medisch Spectrum Twente, Enschede, The Netherlands

**Keywords:** Atrial fibrillation, Pulmonary vein isolation, Ablation, Cryoballoon

## Abstract

**Aims:**

Cryoballoon pulmonary vein (PV) isolation in patients with atrial fibrillation has proven to be effective in short-term and long-term follow-up. To visualise the PV anatomy, pre-ablation contrast pulmonary venography is commonly performed. Three-dimensional (3D) computed tomography (CT) overlay is a new technique creating a live 3D image of the left atrium by integrating a previously obtained CT scan during fluoroscopy. To evaluate the benefits of 3D CT overlay during cryoballoon ablation, we studied the use of 3D CT overlay versus contrast pulmonary venography in a randomised fashion in patients with paroxysmal atrial fibrillation undergoing cryoballoon PV isolation.

**Methods and results:**

Between October 2012 and June 2013, 30 patients accepted for PV isolation were randomised to cryoballoon PV isolation using either 3D CT overlay or contrast pulmonary venography. All patients underwent a pre-procedural cardiac CT for evaluation of the anatomy of the left atrium (LA) and the PVs. In the 3D CT overlay group, a 3D reconstruction of the LA and PVs was made. An overlay of the CT reconstruction was then projected over live fluoroscopy. Patients in the contrast pulmonary venography group received significantly more contrast agent (77.1 ± 21.2 cc vs 40.1 ± 17.6 cc, *p* < 0.001) and radiation (43.0 ± 21.9 Gy.cm2 vs 28.41 ± 11.7 Gy.cm2, *p* = 0.04) than subjects in the 3D CT overlay group. There was no difference in total procedure time, fluoroscopy time and the amount of cryoapplications between the two groups.

**Conclusion:**

The use of 3D CT overlay decreases radiation and contrast dye exposure and can assist in guiding cryoballoon-based PV isolation.

## Introduction

Atrial fibrillation (AF) is the most common arrhythmia, affecting approximately 1.5–2% of the general population [1]. In 1998, the pulmonary veins (PVs) were identified as potential targets for the invasive treatment of AF [2].

PV isolation is an effective treatment for patients with symptomatic paroxysmal AF and recommended in international guidelines [1]. Ablation success rates at 12 months range between 66 and 89% [3–5]. However, radiofrequency ablation requires tedious point-to-point delivery of multiple applications to isolate PVs [6]. As a result, more circular ablation catheters have been developed facilitating PV isolation. Ablation using a cryoballoon has been proven effective in short and long-term follow-up, with equal efficacy and safety, compared with the conventional radiofrequency approach [6–10].

Pre-procedural contrast pulmonary venography is commonly performed to visualise the PV anatomy. Fluoroscopy is inherently associated with significant radiation exposure for both the patient and operator. Improved visualisation using iodine-based contrast agents is dose-dependent related to acute and chronic kidney failure. Several techniques have been evaluated to optimise the visualisation during the cryoablation procedure, such as the use of real-time three-dimensional oesophageal echocardiography [11].

Three-dimensional (3D) computed tomography (CT) overlay creates a live 3D image during the procedure by integrating fluoroscopy with a previous CT scan or newly taken rotational angiographic 3D images of the left atrium (LA) and it has been shown to assist in radiofrequency-based PV isolation [12]. 3D CT overlay can facilitate optimal positioning of the cryoballoon and reduce contrast medium use and radiation exposure. Better positioning of the balloon can decrease the necessity for additional application(s) during PV isolation, using extra balloon or catheter cryoapplications to achieve PV isolation. To evaluate the benefits of 3D CT overlay during cryoballoon ablation, we studied the use of 3D CT overlay versus contrast pulmonary venography in a randomised fashion in consecutive patients with paroxysmal AF undergoing cryoballoon PV isolation.

## Methods

Patients were eligible for enrolment in the study when they were accepted for percutaneous PV isolation for paroxysmal AF, as defined in the current guidelines [1]. The exclusion criteria were as follows: (1) Patients with persistent AF, as defined in the current guidelines [13]; (2) A left atrial diameter of more than 50 mm in the parasternal long axis on transthoracic echocardiography; (3) Previous pulmonary vein isolation ablation (epicardial or endocardial); (4) Previous cardiac surgery; (5) Significant valvular disease present on echo (mitral or aortic valve regurgitation above grade 2, moderate to severe mitral or aortic stenosis); (6) Concomitant cardiac surgery needed; (7) Left ventricular ejection fraction <40%; (8) Hypertrophic (obstructive) cardiomyopathy or dilated cardiomyopathy defined as an ejection fraction <40%; (9) Pregnancy; (10) Myocardial infarction within the previous 3 months; (11) AF secondary to electrolyte imbalance, thyroid disease, other reversible or non-cardiovascular causes for AF. Patients were recruited in the outpatient clinic and included in the study after signed informed consent was obtained.

After inclusion, patients were randomised to cryoballoon PV isolation using 3D CT overlay or contrast pulmonary venography. All patients underwent a pre-procedural cardiac CT for evaluation of the anatomy of the LA and the PVs. If the patient’s PV anatomy was not suitable for cryoballoon ablation, the patient was excluded. Patients were also excluded if the PVs could not be isolated with the cryoballoon alone during the procedure.

### Sample size calculation

To reach statistical significance of 20% difference between the two groups regarding fluoroscopy time and contrast medium, we used the following parameters for power calculation: alpha = 5%; power = 80%; and assuming equal standard deviations in each group. Requiring 47 patients for fluoroscopy time and 17 for contrast medium. An interim analysis would be performed after 30 inclusions in consultation with the medical research ethics committee.

### Pre-procedural cardiac CT

The pre-procedural contrast-enhanced cardiac CT was performed using a 64 multi-slice scanner (Toshiba Aquillion 64, Tokyo, Japan). Images were obtained at 120 kV and 300 mAs. Rotation time was 0.4 ms. The thickness of the reconstructed image slices was 0.3 mm. During a 20-second end/expiratory breath hold, 80 ml contrast (Visipaque 320, GE Healthcare A.S., Oslo, Norway) was injected. An ECG-triggered scan was timed at 50% of the average inter-beat (RR) interval.

### Ablation procedure

Two electrophysiologists (MS and JvO) with extensive experience in cryoballoon ablation performed the procedures. All patients were on oral vitamin-K antagonists with the international normalised ratio between 2.5 and 3.5. Vitamin K antagonists were continued during the procedure [14]. All procedures were performed under general anaesthesia and arterial blood pressure was continuously monitored. Venous access was obtained from the right and left femoral vein. A diagnostic catheter (EP XT CS 4p, BARD Medical Inc., GA, USA) was positioned in the coronary sinus for stimulation of the LA. The LA was accessed by a transseptal puncture with a Brockenbrough needle monitored by intracardiac echocardiography (St. Jude Medical, MN, USA), first with a SL-O sheath (St. Jude Medical, MN, USA), changed over a 0.32 F wire to a steerable 12 F sheath (Flexcath, Medtronic Inc., MN, USA). During the procedure, heparin was given to achieve an activated clotting time of >350 s. The use of a 23 or 28 mm balloon was based on the PV diameters.

### 3D CT overlay group

**Fig. 1:**
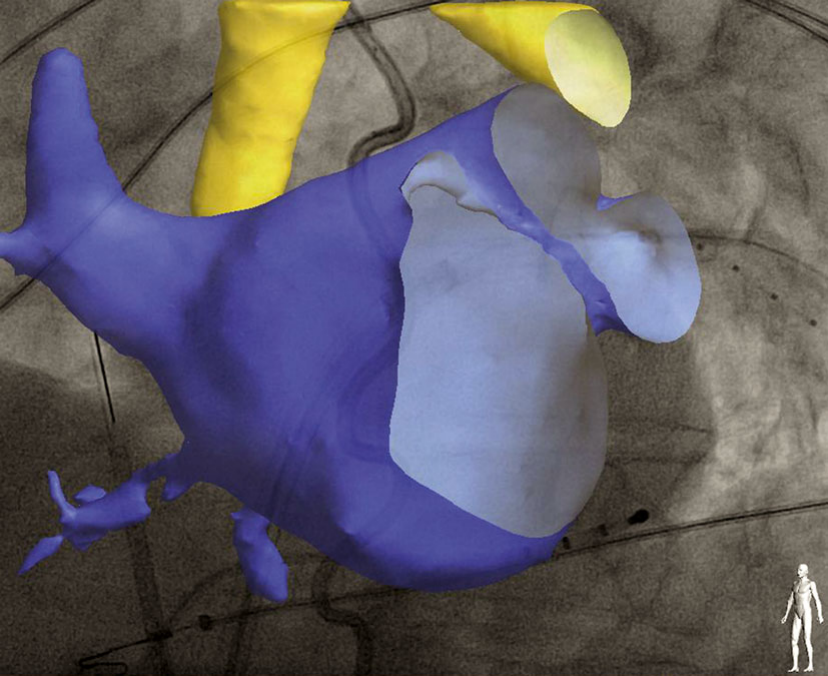
Example of a left atrial 3D CT overlay on the live fluoroscopy. The Achieve catheter is inserted into the left superior PV. A 4-pole catheter is placed in the coronary sinus

Before the start of the procedure, the CT images were imported into the EP Navigator workstation (Philips Medical Systems, Best, the Netherlands) to create a 3D digital reconstruction of the anatomy of the LA and the PVs as previously described [12]. An automated reconstruction tool was used. Manual correction tools were used for optimisation of this reconstruction. An overlay of the CT reconstruction was then applied over live fluoroscopy and registration was performed using anatomical landmarks, catheter positions and contrast boluses in both superior PVs. Validation of the correct registration was performed in anteroposterior and lateral views (Fig. 1).

### Contrast pulmonary venography group

After transseptal puncture, a 7 French NIH catheter (Cordis, Miami Lakes, FL, USA) was introduced and contrast was delivered to each single PV. High definition cinematographic images of the PVs were made in left and right anterior oblique projections.

### Procedure

We used the Arctic Front Advance cryoballoon (Arctic Front Advance, Medtronic Inc., MN, USA). The 28-mm balloon was used in 21 patients. A multipolar catheter (Achieve, Medtronic Inc., MN, USA) was inserted through the inner lumen of the balloon to assess PV signals before, during and after the ablation and to guide the positioning of the balloon, after which the total assembly was introduced into the LA. After inflation, the cryoballoon was advanced to occlude the PV and a contrast bolus was administered to confirm total occlusion. In general, two consecutive applications were delivered for each PV, varying from 180 to 240 s, depending on temperatures reached. During isolation of the right PVs, the right phrenic nerve was continuously stimulated by a catheter placed in the superior vena cava. If the diaphragm excursions diminished during cryoablation, the ablation was immediately stopped using the double stop technique [15]. Bidirectional block was confirmed for each vein. After a 30-minute waiting period, all four PVs were checked for reconduction and adenosine was administered to reveal dormant PV potentials.

### Radiation dose

Effective dose is a convenient quantity to estimate the stochastic risk of radiation applied to patients in interventional procedures [16]. Radiation of cardiac CT was measured as dose length product. The dose length product was converted to the effective dose by using a conversion factor of 0.014 mSv Gy1 cm1 [17]. Radiation of fluoroscopy was measured in dose area product (DAP). The DAP was converted to the effective dose by using a conversion coefficient of 0.188 mSv Gy1 cm2 [18]. For the 3D CT overlay group, the main effective dose was calculated by adding the previously mentioned conversion indices for the cardiac CT and radiation of fluoroscopy.

### Procedure time, number of applications, radiation exposure and contrast agent

The total procedure time was defined as the total time between the first puncture of the femoral vein and the end of the procedure. The following were documented: (1) The time from the first puncture of the femoral vein to the transseptal puncture; (2) The total number of cryoballoon applications; (3) The total fluoroscopy time; (4) The total radiation dose; and (5) The total amount of contrast agent used. We registered the total amount of contrast used for any kind of image enhancement and for determination of the grade of occlusion during cryoablation. We therefore reported contrast usage in both groups for any reason other than visualising the PVs. In the pulmonary venography group, the amount of contrast agent used for PV visualisation alone was also registered.

### Follow-up

After the intervention, patients were scheduled for four outpatient clinic visits at 1, 3, 6 and 12 months. Antiarrhythmic drugs were withdrawn after a stabilisation period of 90 days after the initial procedure and post-procedural experienced AF burden. Prior to the outpatient clinic visits at 3, 6 and 12 months, a 7-day auto-triggered event recording was performed using a Vitaphone recorder (Vitaphone GmbH, Mannheim, Germany) [19, 20]. An episode of AF is defined as an episode of at least 30 s’ duration.

### Statistical analysis

Results were analysed using the SPSS 17.0 software (SPSS Inc., Chicago, IL, USA). Independent samples T‑test was used for numerical normally distributed data and the *χ*2 test was used for categorical variables. *P*-value *<*0.05 was considered statistically significant.

## Results

From October 2012 until June 2013, 30 patients were enrolled in the study. Two patients were excluded because PV isolation could not be completed with the cryoballoon alone and additional radiofrequency applications during the same procedure were needed to achieve PV isolation (equal distribution in both groups). Table 1 shows the baseline characteristics. Mean age was 57.3 ± 8.9 years. Hypertension was present in six subjects (21.4%). Mean CHADS2-VASc score was 0.8. Mean corrected left atrial end/diastolic volume was 25.1 ± 7.2 cc/m2. Anticoagulation was used by nine patients (32.1%) at the time of randomisation. No significant differences were found between the two operators. Table 2 shows radiation doses and amounts of contrast. Patients in the contrast pulmonary venography group received significantly more contrast agent than subjects in the 3D CT overlay group. Furthermore, patients in the direct fluoroscopy group received more radiation, as expressed in the DAP-value. There were no differences in fluoroscopy time, number of cryoapplications and total procedure time between the two groups. No statistical differences were found with *p* < 0.05 using an intention-to-treat analysis involving all randomised patients.

**Table 1:** Baseline characteristics

Variable	Total *n* = 28	3D CT overlay *n* = 14	Pulmonary Venography *n* = 14	*p*-value
Age (years)	57.3 ± 8.9	58.2 ± 7.6	56.5 ± 10.3	ns
Gender (male)	20 (71.4%)	9 (64.3%)	11 (78.6%)	ns
CHADS_2_-VASc score	0.8 ± 1.1	0.7 ± 0.7	0.9 ± 1.4	ns
Hypertension	6 (21.4%)	3 (21.4%)	3 (21.4%)	ns
Smoking	8 (28.6%)	1 (7.1%)	7 (50.0%)	0.011
Diabetes	0 (0%)	0 (0%)	0 (0%)	–
AF family history <65 years	9 (32.1%)	3 (21.4%)	6 (42.9%)	ns
CAD	0 (0%)	0 (0%)	0 (0%)	–
CHF	0 (0.0%)	0 (0.0%)	0 (0.0%)	–
Endurance sports	3 (10.7%)	2 (14.3%)	1 (7.1%)	ns
OSAS	1 (3.6%)	0 (0.0%)	1 (7.1%)	ns
TIA/CVA	1 (3.6%)	0 (0.0%)	1 (7.1%)	ns
Flutter	3 (10.7%)	2 (14.3%)	1 (7.1%)	ns
Anticoagulants	9 (32.1%)	6 (42.9%)	3 (21.4%)	ns
No ECV	1.2 ± 1.9	1.0 ± 1.7	1.4 ± 2.1	ns
BMI	26.8 ± 3.0	26.3 ± 2.6	27.2 ± 3.4	ns
LA volume (cc/m2)	25.1 ± 7.2	25.1 ± 6.2	25.1 ± 8.3	ns

Data are expressed in mean ± SD or absolute number and percentage

*AF* Atrial fibrillation, *CAD* Coronary artery disease, *CHF* Congestive heart failure (Ejection Fraction <40%), *OSAS* Obstructive sleep apnoea syndrome, *TIA* Transient ischaemic attack, *CVA* Cerebral vascular attack, *ECV* Electrical cardioversion, *BMI* Body mass index, *LA* Left atrium, *NS* Not significant

**Table 2:** Procedure results

Variable	Total *n* = 28	3D CT overlay *n* = 14	Pulmonary Venography *n* = 14	*p*-value
Total Contrast used (cc) – Without PV angio	58.6 (±26.9) 36.8 (±15.9)	40.1 (±17.6) 40.1 (±17.6)	77.1 (±21.2) 33.4 (±13.8)	<0.001 ns
DAP (Gy.cm2)	35.69 (±18.8)	28.41 (±11.7)	43.0 (±21.9)	0.04
Effective dose procedure (mSv)	6.71 (±3.5)	5.34 (±2.2)	8.08 (±4.1)	0.04
Fluoroscopy time (min)	29.4 (±9.8)	28.8 (±11.2)	30.1 (±8.4)	ns
Needle-TSP (min)	16.5 (±8.4)	14.7 (±3.6)	18.2 (±11.3)	ns
Procedure time (min)	110.1 (±28.5)	113.2 (±36.7)	106.9 (±17.8)	ns
Cryo-applications per procedure	9.3 (±2.2)	9.9 (±2.7)	8.7 (±1.3)	ns

Data are expressed in mean ± SD

*PV angio* Pulmonary vein angiography, *DAP* Dose area product, *TSP* Transseptal puncture, *NS* Not significant

### Complications

Complications were present in three patients (10.7%). In one patient (direct fluoroscopy group), the procedure was complicated by permanent vagal nerve injury, resulting in gastric paralysis. One patient (3D CT overlay group) had transient gastric paralysis. One patient (direct fluoroscopy group) suffered from transient right hemidiaphragm paralysis. After the occurrence of vagal nerve injuries, a thermoprobe (SensiTherm, St. Jude Medical, MN, USA) was introduced in our PV procedures to monitor oesophageal temperatures during PV isolation, after which this complication did not longer occur [21].

### Clinical outcome

At a mean follow-up of 11.9 ± 3.9 months (median 12 months), the success rate without continuation of antiarrhythmic therapy was 78.6% (22 patients). No difference in recurrence of AF was detected between 3D CT overlay and the contrast group (12 vs 10 *p* = 0.55). One patient was lost to follow-up. Four patients were treated in a second procedure using radiofrequency ablation with touch-up of isolation gaps. Successful isolation of all PVs could not be reached in 1 patient, for which a successful video-assisted thoracoscopy PV isolation was performed. Prolonged follow-up for 9.6 ± 3.5 months (median 10 months) showed no recurrence.

## Discussion

This study shows the feasibility of 3D CT overlay in PV isolation using the cryoballoon and demonstrates significantly less radiation and use of contrast in comparison to contrast pulmonary venography. The use of 3D CT overlay has several benefits over direct fluoroscopy. As the 3D CT overlay image supplies a 3D navigation map, manoeuvring and placing the cryoballoon becomes more straightforward. This has also been demonstrated for 3D transoesophageal echocardiography [11]. The relative difference in radiation dose and radiation time can be explained by the fact that, for cinematographic images of the PV angiograms, a higher resolution was needed.

Contrast-induced acute kidney injury is an important complication of the use of iodinated contrast media, which accounts for a significant number of cases of hospital-acquired acute kidney injury [22]. As PV isolation is increasingly becoming standard therapy for patients with AF, it is essential to optimise patient safety. We realise that a CT scan requires radiation and contrast agent as well. In our study population, a pre-procedural CT scan was performed on all subjects. The amount of contrast agent (Visipaque 320) was 80 or 90 cc. Main dose length product of the CT scan in our study population was 841.3 ± 255.5 mGy.cm. Using the conversion factor of 0.014 mSv Gy1 cm1 [17], this results in a main effective dose of 11.8 mSv. The combined effective dose in the 3D CT overlay group is still lower in comparison to that of the direct fluoroscopy group.

However, ideally the radiation and contrast agent necessary for the CT scan is avoided as well. This can be done by using a 3D overlay from magnetic resonance imaging (MRI). Recently, we successfully used a 3D MRI overlay in PV isolation with the cryoballoon in two patients. Although MRI was not used in this study, it could replace the CT scan for the 3D overlay and even further reduce total radiation and contrast dye exposure.

Complications were present in three (10.7%) patients, two minor complications and one major. In all three cases, the 23-mm balloon was used. Andrade et al. demonstrated that the smaller balloon is associated with a high incidence of phrenic nerve palsy [7]. We report two cases of va﻿gal nerve injury. Kuwahara et al. reported peri-oesophageal nerve injury as a complication of PV isolation with radiofrequency energy in 11/3695 patients [23]. Recent reports also demonstrate an increased incidence of oesophageal thermal lesions using the second-generation 28-mm cryoballoon [24]. We introduced continuous measurement of luminal oesophageal temperature after this complication, after which no vagal nerve injuries occurred. The study was terminated prematurely due to a significant difference in radiation dosage at interim analysis. Therefore, the study was underpowered with regard to the contrast dosage determined by our initial power analysis.

## Conclusion

The use of 3D CT overlay has several benefits over direct fluoroscopy. As the 3D CT overlay image supplies a 3D navigation map, manoeuvring and placing the cryoballoon becomes more straightforward.

The present study shows that the use of 3D CT overlay has the potential to reduce radiation dose and exposure to contrast dye in cryoballoon-based PV isolation. Even though the study population is limited, the results show a clear benefit of this technique. We expect an additional advantage of 3D MRI overlay with respect to radiation dose. More research into this technique is necessary and will be effectuated.
