# Erratum to: Use of three-dimensional computed tomography overlay for real-time cryoballoon ablation in atrial fibrillation reduces radiation dose and contrast dye

**DOI:** 10.1007/s12471-017-1013-0

**Published:** 2017-06-19

**Authors:** B. Oude Velthuis, M. M. D. Molenaar, H. G. Reinhart Dorman, J. Y. Stevenhagen, M. F. Scholten, J. van der Palen, J. M. van Opstal

**Affiliations:** 1 Thorax Centre Twente, Medisch Spectrum Twente, Enschede, The Netherlands; 2 Department of Cardiology, Bravis Ziekenhuis, Roosendaal, The Netherlands; 3 Department of Research methodology, Methods and Data Analysis, University of Twente, Enschede, The Netherlands; 4 Medical School Twente, Medisch Spectrum Twente, Enschede, The Netherlands

**Erratum to: Neth Heart J (2017) DOI: 10.1007/s12471-017-0962-7**

In the version of the article originally published online the name of the second author was incomplete and should have read: M.M.D. Molenaar

